# Supplementary material for: Purine metabolism regulates DNA repair and therapy resistance in glioblastoma
Source: Nat Commun. 2020 Jul 30;11:3811. doi: 10.1038/s41467-020-17512-x (PMC7393131; doi:10.1038/s41467-020-17512-x)
Supplement: Supplementary file 1 — Supplementary Information [file 41467_2020_17512_MOESM1_ESM.pdf]

SUPPLEMENTARY INFORMATION

Purine metabolism regulates DNA repair and therapy resistance in glioblastoma

Weihua Zhou[1]†, Yangyang Yao[1,2]†, Andrew J. Scott[1,3]†, et al.

Supplementary Methods

1. Cell culture and reagents: the details of the origins of the GBM cell lines used in our study are listed in supplementary Table 1. HF2303 primary neurospheres, which were originally described by Dr. Tom Mikkelsen at Henry Ford Hospital (Detroit, MI) [1], were a kind gift from Dr. Alnawaz Rehemtulla and MSP12 was a gift from Drs. Pedro Lowenstein and Maria Castro. Cell line authentication was performed by the originating cell line repositories and then used immediately upon receipt. Cell lines were re-authenticated using short tandem repeat profiling if they had been using for longer than 1 year. Mycoplasma test (Cat# LT07-418, Lonza) were performed monthly in our lab.

All immortalized GBM adherent cells were cultured in DMEM (Cat# 11965-092, Gibco) supplemented with 10% FBS (Cat# S11550, ATLANTA biologicals), 100 μg/mL Normocin (Cat# ant-nr-1, InvivoGen) and 100 U/mL Penicillin-Streptomycin-Glutamine (Cat# 10378-016, Gibco). Primary patient-derived GBM neurospheres (HF2303 and MSP12) were cultured in DMEM-F12 (Cat# 10565-018, Gibco) supplemented with B-27 supplement (Cat# 17504-044, Thermofisher), N2 supplement (Cat# 17502-048, Thermofisher), 100 U/mL Penicillin-Streptomycin (Cat# 15140122, Thermofisher), 100 μg/mL Normocin (Cat# ant-nr-1, InvivoGen), and 20 ng/mL epidermal and fibroblast growth factors (Cat# AF-100-15 and #100-18B, PeproTech). For hypoxanthine deprivation assay, U87 MG cells were cultured in Ham's F-12 medium (Cat# C837J99, Thomas Scientific) containing 10% dialyzed FBS (Cat# A3382001, Invitrogen), with (~30 μM) or

without hypoxanthine.

Individual nucleosides, including uridine (Cat# U3003), thymidine (Cat# T1895), cytidine (Cat# C4654), adenosine (Cat# A4036), guanosine (Cat# G6264), teriflunomide (Cat# SML0939), and MPA (Cat# M5255), were purchased from Sigma. The other reagents used in the study include Acummax (Cat# AM-105, Innovative cell technologies Inc), Amide-15N L-glutamine (Cat# NLM-557-1, Cambridge Isotope Laboratories), MMF (Cat# S1501, Selleckchem), and pooled nucleosides (Cat# ES-008-D, Millipore). Pooled nucleosides were used at a working concentration of 8x, which yielded final concentrations as follows: cytidine 0.0584 g/L (~240.12 μM), guanosine 0.068 g/L (~240.08 μM), uridine 0.0584 g/L (~240.12 μM), adenosine 0.064 g/L (~239.48 μM) and thymidine 0.0192 g/L (~79.26 μM).

The luciferase-IRES-GFP bicistronic expression cassette (LeGO-iG2-FLuc vector) was kindly provided by Dr. Samuel K. McBrayer [2]. The lentiviruses harboring fluc (lenti-LEGO-Ig2-fluc-IRES-GFP-VSVG) were made in the University of Michigan Vector Core.

2. CCLE and Gene Set Enrichment Analysis: relationships between RT-resistance and transcript levels were determined across 23 cell lines using simple least-squares linear regression between empirically determined Dmid (Fig. 1A) and gene expression as determined by the Broad Institute Cancer Cell Line Encyclopedia (CCLE) version 2012-10-18 [3]. For genes of interest (*IDH1*, *IDH3a* and *GLUL*), a likelihood ratio test was performed between a simple linear regression model with Dmid as dependent variable and mRNA expression as independent variable plus the

intercept and a null model with only the intercept as independent variable. Its significance was assessed using the p-value corresponding to the test Chi-squared statistic, which were adjusted for multiple testing using the Benjamini-Hochberg correction. Genes were then rank-ordered by correlation coefficient and gene set enrichment analysis (GSEA, Supplementary Figs. 1F-G) was performed to identify the top gene sets correlated with RT-resistance and RT-sensitivity [4].

3. Establishment of HPRT1 stable cell lines: MSP12 was transfected with the lentiviral particles of pLKO.1-shRNA of HPRT1 (Cat# TRCN0000035050, Sigma) or non-target shRNA (Cat# SHC016, Sigma) and selected with puromycin at 2 μg/mL for three days. Pooled cells were harvested and knockdown confirmed by immunoblotting.

4. Immunofluorescence: immortalized or primary GBM cells were plated and treated with indicated conditions. Cells were then collected and fixed at indicated time points using 4% paraformaldehyde. Primary neurospheres were mixed with the Histogel (Cat# HG-4000-012, Thermo Scientific) and transferred to tissue cassettes for paraffin embedding, section and staining. γ-H2AX foci were detected with mouse monoclonal antibody anti-phospho-Histone H2AX (1:1000 dilution; Cat #05-636, Millipore) and goat anti-mouse IgG cross-adsorbed secondary antibody, alexa fluor 594 (1:2000 dilution; Cat# A-11005, Invitrogen). DNA was stained with DAPI. γ-H2AX foci were scored for each condition in more than 100 cells of immortalized GBM cells and more than 15 spheres of primary GBM cells. The foci threshold, which is used to define a positive cell, was 10 for immortalized cells and 3 for

primary GBM neurospheres.

5. Flow cytometry analysis: for cell cycle analysis, cells were harvested and fixed with pre-cooled 70% ethanol overnight, and staining with propidium iodide/RNase A staining buffer (Cat# 550825, BD Bioscience) in the dark at room temperature for 15 min before acquiring data on flow cytometer [5]. Data were analyzed on a FACScan flow cytometer (Becton Dickinson) with FlowJo software 7.6 (Tree Star). For γ-H2AX analysis, samples were incubated with a mouse monoclonal antibody anti-phospho-Histone γ-H2AX (1:500 dilution; Cat #05-636, Millipore) and with a FITC conjugated anti-mouse secondary antibody (1:2000 dilution; Cat# A-11005, Invitrogen), followed by staining with propidium iodide/RNase A to assess total DNA content. Data were analyzed on a FACScan flow cytometer (Becton Dickinson) with FlowJo software 7.6 (Tree Star) [6].

6. Celltiter-Glo cell viability assay: MSP12 or HF2303 primary GBM cells were dissociated and plated into 6-well plates and allowed to form spheres. 3-4 days post-plating, neurospheres were treated with nucleosides and/or MPA, followed by irradiation. Twenty-four hours after RT, cells were disassociated into single cells and replated into 96-well plates with around 2000 cells per well. After growing for 7 to 10 days, cell viability as detected using CellTiter-Glo® 3D Reagent (Cat# G9682, Promega) following the manufacturer’s protocol.

7. Alkaline comet assay: GB-1 and DBTRG-05MG cells were plated and treated with indicated conditions at different time points. Single-cell gel electrophoretic comet assays were performed under alkaline conditions [7]. Briefly, cells were

combined with 1% LM Agarose (Cat# IB70051, IBI SCIENTIFIC) at 40 ºC at a ratio of 1:10 (vol/vol) and immediately pipetted onto slides. For cellular lysis, the slides were immersed in lysis solution (Cat# 4250-050-01, R&D SYSTEMS) overnight at 4 °C in the dark, followed by washing in alkaline unwinding solution (200 mM NaOH, 1 mM EDTA, pH > 13) for 30 min. Then, the slides were immersed in alkaline electrophoresis solution (200 mM NaOH, 1 mM EDTA, pH > 13) to be subjected to electrophoresis at 21 V for 30 min and stained in 2.5 μg/mL propidium iodide (Cat# P3566, Invitrogen) for 20 min. All images were taken with a fluorescence microscope and analyzed by Comet Assay IV software (Perceptive Instruments). For quantification, the tail moment, a measure of both amount and distribution of DNA in the tail, was used as an indicator of DNA damage. Comet-positive cells were scored in random fields of cells, with more than 100 cells for each experimental condition.

8. Clonogenic survival assay: Cells were irradiated with indicated doses and treated with or without compounds, following by replating at clonal density. Plates were stained and colonies containing > 50 cells were counted after 10 to 14 days of growth. The RT enhancement ratios were calculated as the ratio of the mean inactivation dose (Dmid) under control conditions divided by the mean inactivation dose under drug-treated conditions[8, 9]. Dmid is defined as the linear area under the linear-quadratic clonogenic survival curve[10]. For sphere-forming assays, enhancement ratios were calculated as the ratio of the GI_{50} in the control condition divided by the GI_{50} in the treated condition.

9. Immunoblotting assay: Xenografts were ground and lysed using RIPA lysis buffer

(Cat# 89900, Thermo Scientific) supplemented with PhosSTOP phosphatase inhibitor (Cat# 04906845001, Roche) and complete protease inhibitor tablets (Cat# 1187358001Roche) [11]. Proteins were detected with antibodies of γ-H2AX (1:1000 dilution; Cat# 05-636, Millipore), HGPRT (1:1000 dilution; Cat# PA5-22281, Thermo Fisher Scientific) and β-Actin (1:5000 dilution; Cat# 4967, Cell Signaling Technology).

10. Immunohistochemical staining: mouse tumors were harvested, fixed in 10% formalin, and embedded in paraffin. Immunohistochemical analyses were performed using the ABC Vectastain Kit (Vector Laboratories) [12]. After deparaffinization, rehydration, antigen retrieval, and blocking, the tumor tissue slides were incubated with primary Ki-67 antibody (1:10000 dilution; Cat# 550609, BD Biosciences) at 4°C overnight. After being incubated with the secondary antibody for 30 min, the tissue slides were stained with DAB (3, 3-diaminobenzidine) and finally counterstained with hematoxylin, dehydrated and mounted.

Supplementary References:

1. Wilson TJ, Zamler DB, Doherty R, Castro MG, Lowenstein PR. Reversibility of glioma stem cells' phenotypes explains their complex in vitro and in vivo behavior: Discovery of a novel neurosphere-specific enzyme, cGMP-dependent protein kinase 1, using the genomic landscape of human glioma stem cells as a discovery tool. Oncotarget **7**, 63020-63041 (2016).

2. McBrayer SK, et al. Transaminase Inhibition by 2-Hydroxyglutarate Impairs Glutamate Biosynthesis and Redox Homeostasis in Glioma. Cell **175**, 101-116 e125 (2018).

3. Barretina J, et al. The Cancer Cell Line Encyclopedia enables predictive modelling of anticancer drug sensitivity. Nature **483**, 603-607 (2012).

4. Subramanian A, et al. Gene set enrichment analysis: a knowledge-based approach for interpreting genome-wide expression profiles. Proc Natl Acad Sci U S A **102**, 15545-15550 (2005).

5. Zhang W, et al. CyclinG1 Amplification Enhances Aurora Kinase Inhibitor-Induced Polyploid Resistance and Inhibition of Bcl-2 Pathway Reverses the Resistance. Cell Physiol Biochem **43**, 94-107 (2017).

6. Parsels LA, et al. PARP1 Trapping and DNA Replication Stress Enhance Radiosensitization with Combined WEE1 and PARP Inhibitors. Mol Cancer Res **16**, 222-232 (2018).

7. Olive PL, Banath JP. The comet assay: a method to measure DNA damage in individual cells. Nat Protoc **1**, 23-29 (2006).

8. Morgan MA, Parsels LA, Kollar LE, Normolle DP, Maybaum J, Lawrence TS. The combination of epidermal growth factor receptor inhibitors with gemcitabine and radiation in pancreatic cancer. Clin Cancer Res **14**, 5142-5149 (2008).

9. Wahl DR, et al. Glioblastoma Therapy Can Be Augmented by Targeting IDH1-Mediated NADPH Biosynthesis. Cancer Res **77**, 960-970 (2017).

10. Fertil B, Dertinger H, Courdi A, Malaise EP. Mean inactivation dose: a useful concept for intercomparison of human cell survival curves. Radiat Res **99**, 73-84 (1984).

11. Zhou W, et al. UBE2M Is a Stress-Inducible Dual E2 for Neddylation and Ubiquitylation that Promotes Targeted Degradation of UBE2F. Mol Cell **70**, 1008-1024 e1006 (2018).

12. Zhou W, et al. Neddylation E2 UBE2F Promotes the Survival of Lung Cancer Cells by Activating CRL5 to Degrade NOXA via the K11 Linkage. Clin Cancer Res **23**, 1104-1116 (2017).

Supplementary Fig. 1

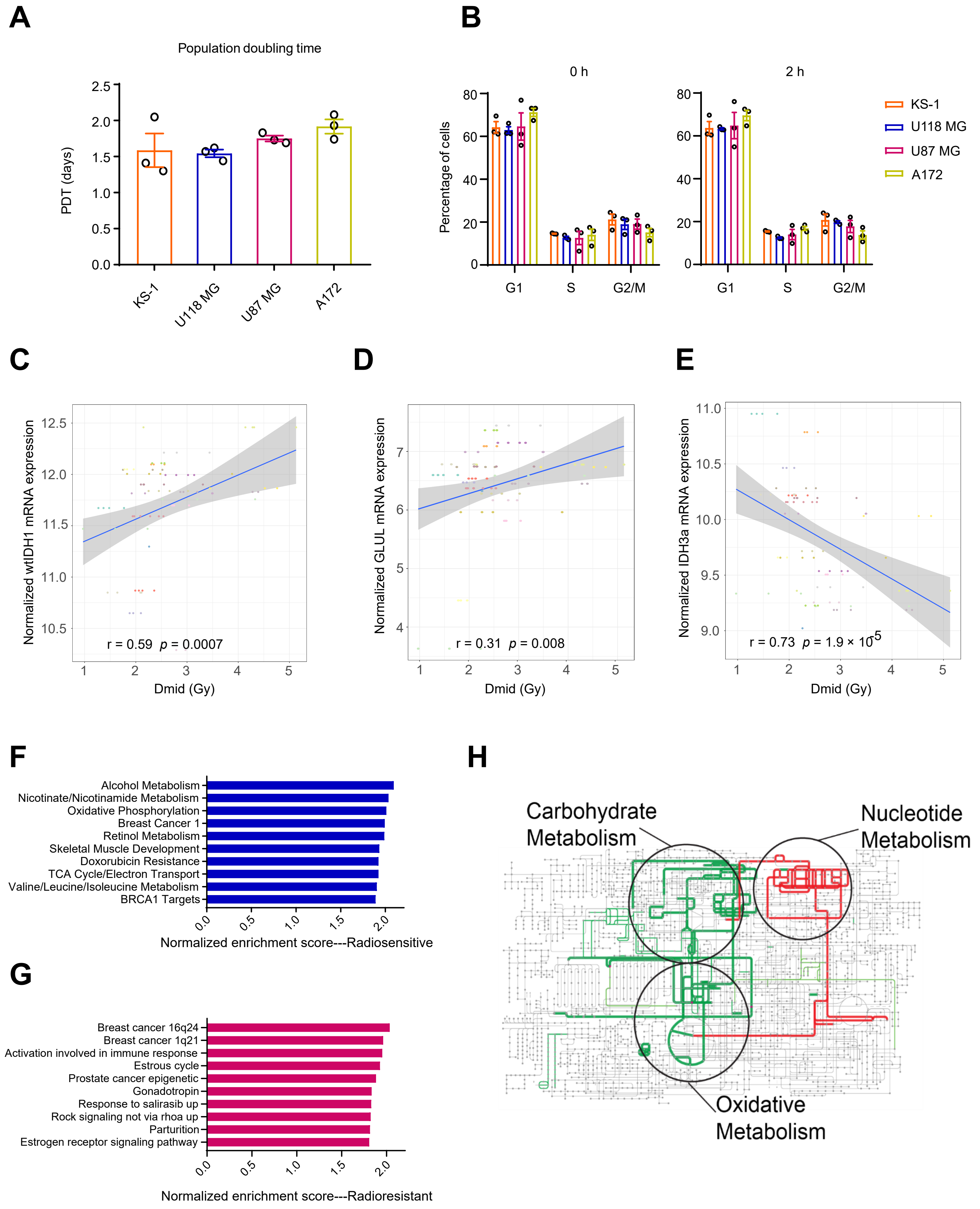

Supplementary Figure Legends

Supplementary Figure 1. Biologic correlates of GBM RT-sensitivity

(A) Indicated GBM cell lines were plated, and cell number were counted at multiple timepoints over the next 8 days to calculate population doubling time (PDT). Data are presented as mean ± SEM from 3 biologic replicates. **(B)** RT-resistant (U87 MG and A172) and RT-sensitive (KS-1 and U118 MG) GBM cell lines were irradiated with 8 Gy, and cells were harvested for flow cytometry cell cycle analysis 2 h post-RT. Data are presented as mean ± SEM from 3 biologic replicates. **(C-E)** Correlation analysis between *IDH1*, *GLUL*,and *IDH3a*, transcript levels and RT-sensitivity of GBMs. The grey shaded regions represent 95% confidence intervals. A likelihood ratio test was performed between a simple linear regression model with Dmid as dependent variable and mRNA expression as independent variable plus the intercept and a null model with only the intercept as independent variable. The test significance was assessed using the *p*-value corresponding to the test Chi-squared statistic. *p*-values were adjusted for multiple testing using the Benjamini-Hochberg correction. The so-computed false-discovery rate (FDR) and the slope coefficient r were reported for each gene. An association between Dmid and mRNA expression was deemed significant if FDR < 0.01. **(F&G)** GSEA analysis of pathway correlates of RT-sensitivity and RT-resistance. **(H)** Pathways with downregulated metabolites that are correlated with RT-sensitivity are shown on a network map of human metabolism (Pearson's correlation $p < 0.08$). Pathways with negative and positive correlation with RT-sensitivity are shown in green and red respectively. Source data are provided as a Source Data file.

Supplementary Fig. 2

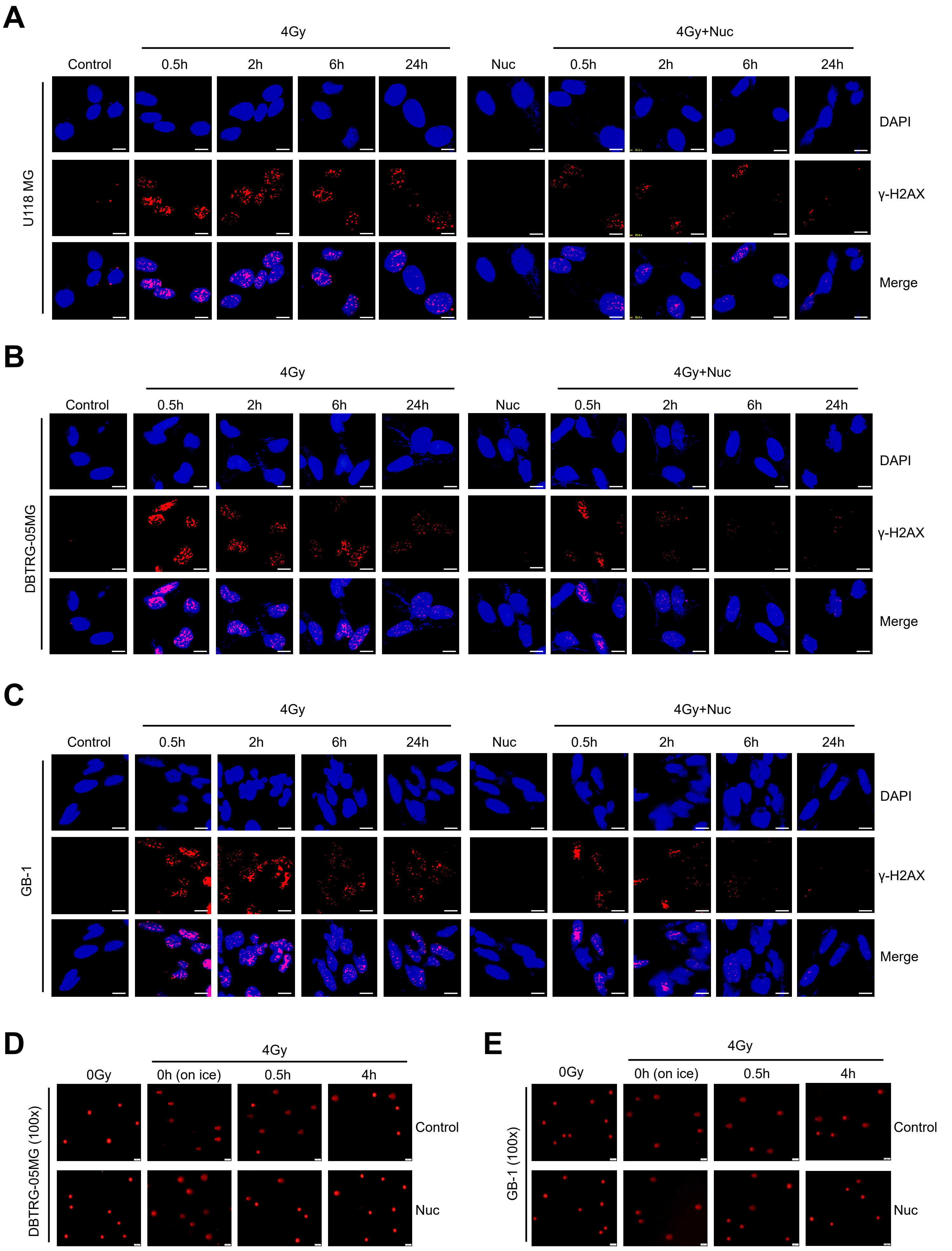

Supplementary Figure 2. Exogenous nucleosides protect RT-sensitive GBMs from RT

(A-C) Representative images of γ-H2AX foci staining in U118 MG (corresponding to Fig. 2E), DBTRG-05MG (corresponding to Fig. 2F) and GB-1 (corresponding to Fig. 2G) cells. Cells were treated with pooled nucleosides, irradiated and fixed for γ-H2AX foci staining at the indicated time points post-RT. Figs. A-C are representative figures from 3 biologic repeats. Scale bar: 50 μm. **(D&E)** Representative images of alkaline comet assay in DBTRG-05MG (corresponding to Fig. 2H) and GB-1 (corresponding to Fig. 2I) cells. DBTRG-05MG or GB-1 cells were plated, treated with exogenous pooled nucleosides, and analyzed by alkaline comet assay at the indicated time points. Fig. D and E are representative figures from 3 (D) or 4 (E) biologic repeats. Scale bar: 100 μm.

Supplementary Fig. 3

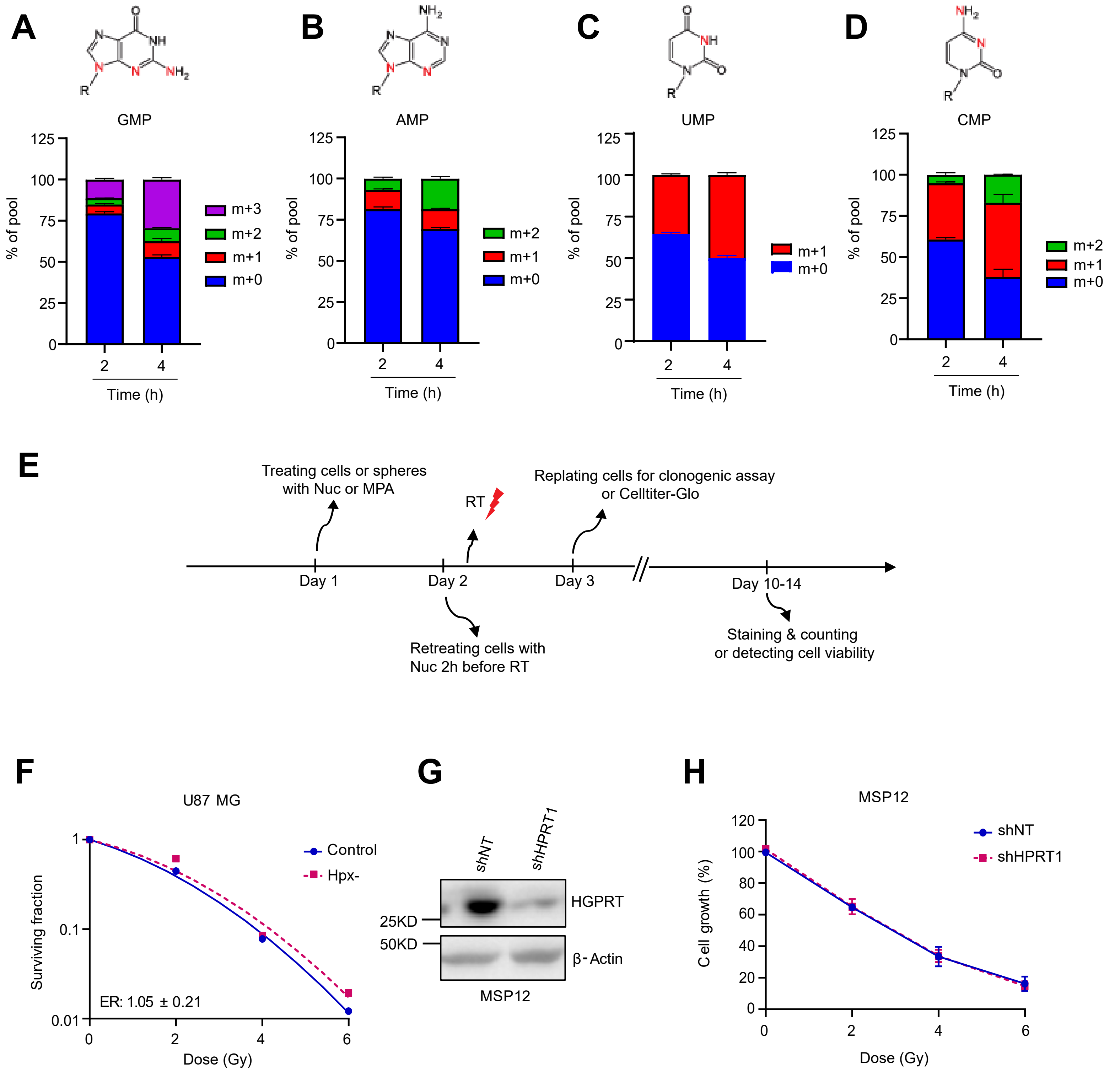

Supplementary Figure 3. ***De novo*** **nucleotide synthesis is active in RT-resistant GBM model and inhibiting purine salvage fails to radiosensitize RT-resistant GBM model**

(A-D) *De novo* nucleotide synthesis is active in RT-resistant GBM cells. U87 MG cells were incubated with DMEM media with 10% dialyzed FBS and 2 mM ^{15}N-amide labeled glutamine for the indicated time (2 h and 4 h). Metabolites were extracted and analyzed by LC/MS. The percentage of each metabolite containing 1 (m+1), 2 (m+2) or 3 (m+3) glutamine-derived nitrogens is plotted. Inset structures show nitrogens in each nucleotide that derive from the glutamine amide. Data are presented as mean ± SD from three biologic replicates. **(E)** A schematic timeline of treatment in RT-resistant cell lines. Cells were treated with pooled nucleosides and/or MPA (10 µM) for 24 h, and retreated with pooled nucleosides 2 h before RT (4 Gy), followed by clonogenic assay or Celltiter-Glo viability assay. **(F)** Hypoxanthine depletion does not impact RT sensitivity in U87 MG cells. U87 MG cells seeded in either control medium (30 µM hypoxanthine) or hypoxanthine-free medium containing 10% dialyzed FBS were irradiated at the indicated doses, plated at clonal density for 10-14 days and then assessed for clonogenic survival. Note: Fig. F is a representative figure from 2 biologically independent experiments, each performed in technical triplicate; Hpx- means hypoxanthine-free. Error bars are mean ± SEM from three technical triplicates. **(G)** Knockdown of HPRT1 was performed in MSP12 neurospheres and confirmed by immunoblotting. Fig. G are representative figures from 3 biologically independent experiments. **(H)** Control (shNT) or HPRT1 knockdown (shHPRT1) MSP12 neurospheres were irradiated with indicated doses, and replated 24 h later in 96-well plates (2000 cells/well) for the CelltiterGlo assay. Curves are a representative experiment that was repeated three separate times (each in technical triplicate). Error bars indicate mean ± SEM from technical triplicates. Source data are provided as a Source Data file.

Supplementary Fig. 4

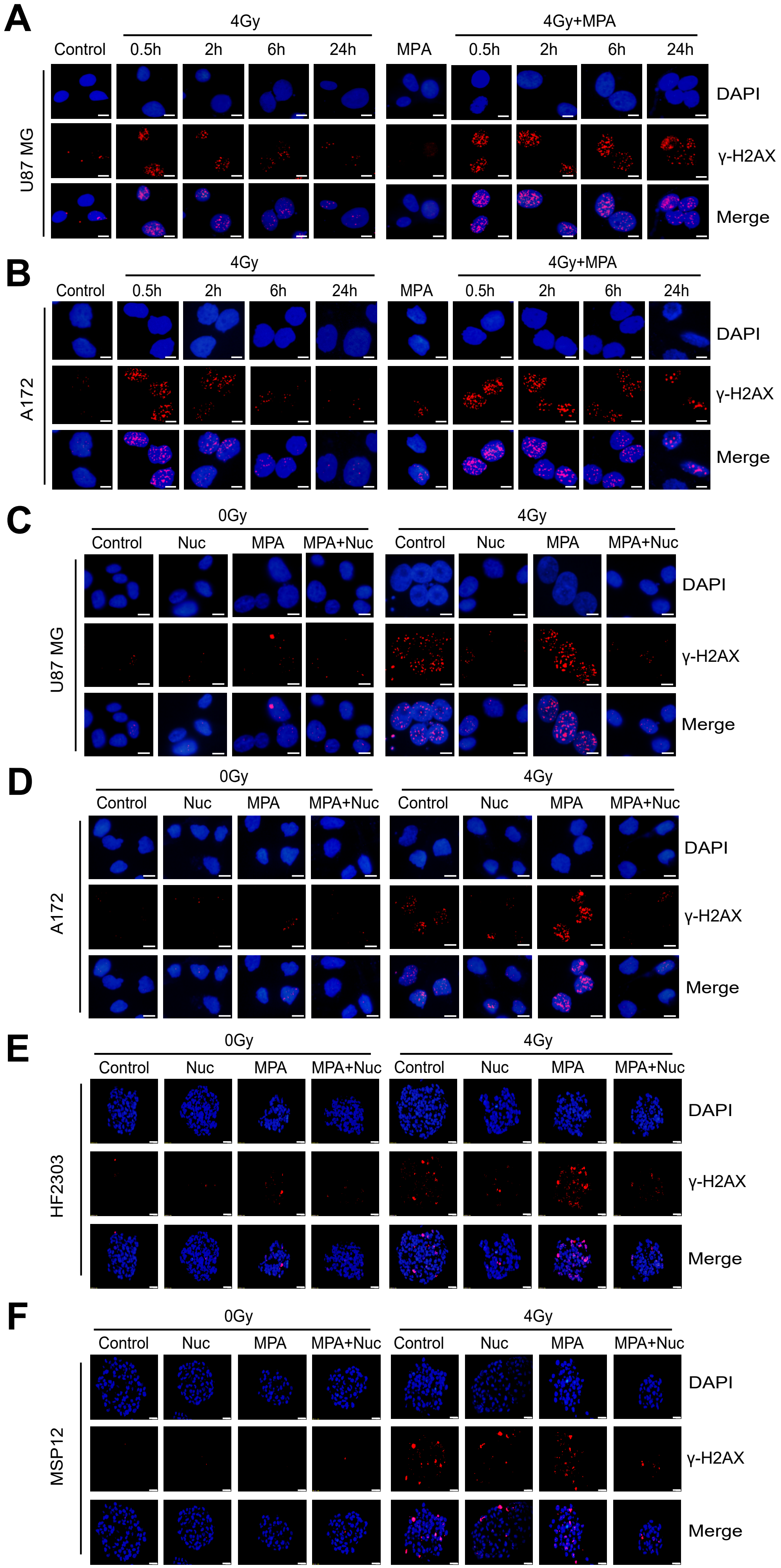

Supplementary Figure 4. Inhibiting *de novo* purine synthesis impairs DNA repair in a nucleoside-dependent fashion

(A&B) Representative images of γ-H2AX foci staining in U87 MG (corresponding to Fig. 4A), and A172 (corresponding to Fig. 4B) cells. Cells were treated with MPA (10 µM) for 24 h and irradiated with 4 Gy, followed by γ-H2AX foci IF staining at indicated timepoints. Fig. A is one representative figure from 3 biologic repeats and Fig. B is one representative figure from 6 biologic repeats **(C-F**) Representative images of γ-H2AX foci IF staining in U87 MG (corresponding to Fig. 4C), A172 (corresponding to Fig. 4D), HF2303 (corresponding to Fig. 4E), and MSP12 (corresponding to Fig. 4F) cells. Cells were treated with indicated conditions and then irradiated, followed by γ-H2AX foci staining 6 h post-RT. Figs. C-F are representative figures from 3 biologic repeats; scale bar: 50 µm.

Supplementary Fig. 5

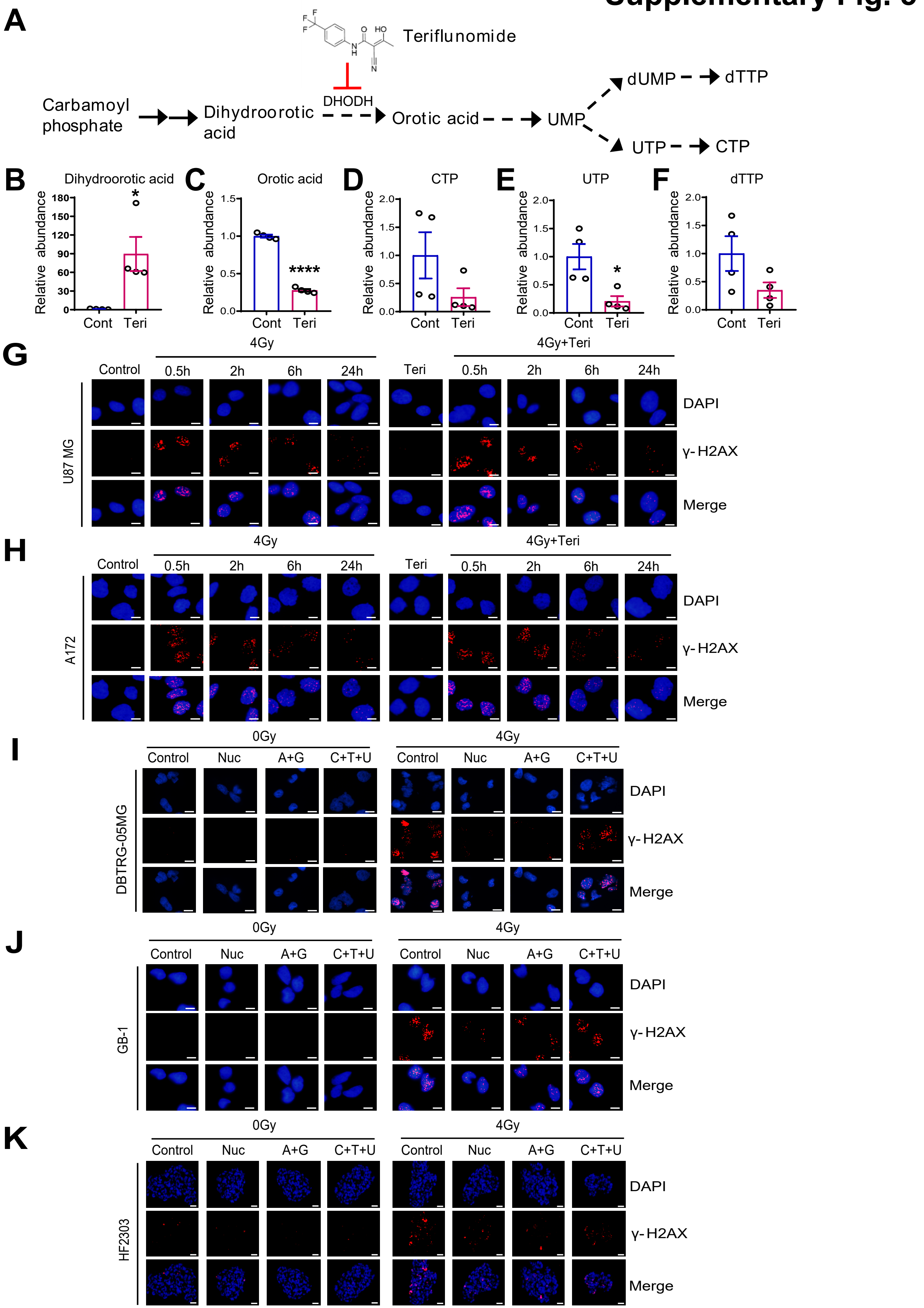

Supplementary Figure 5. Modulating pyrimidine pools has minimal effects on DNA repair and RT-resistance in GBM

(A) A schematic diagram of *de novo* pyrimidine synthesis. DHODH: dihydroorotate dehydrogenase; UMP: uridine monophosphate; dUMP: deoxyuridine monophosphate; dTTP: deoxythymidine triphosphate; UTP: uridine triphosphate; CTP: cytidine triphosphate. **(B-F)** U87 MG cells were treated with 20 μM teriflunomide for 24 h, and then harvested for targeted LC/MS assay. Data are presented as mean ± SEM from four biologic replicates. *p*-values are 0.0177 (Fig. B), <0.0001 (Fig. C), and 0.0172 (Fig. E); *, $p < 0.05$; ****, $p < 0.0001$. The *p*-values indicated were obtained by two-tailed unpaired student t test. **(G&H)** Representative images of γ-H2AX foci IF staining in U87 MG (corresponding to Fig. 5C), and A172 (corresponding to Fig. 5D) cells. Cells were treated with teriflunomide (20 μM) for 24 h, and irradiated (4 Gy), and harvested at indicated time points post-RT for γ-H2AX foci staining. **(I-K)** Representative images of γ-H2AX foci in DBTRG-05MG (corresponding to Fig. 5E), GB-1 (corresponding to Fig. 5F), and HF2303 (corresponding to Fig. 5G) cells. Cells were treated with indicated conditions and then irradiated, followed by γ-H2AX foci staining 6 h post-RT. Note: Figs. G, H and K are representative figures from 3 biologic repeats; Figs. I and J are representative figures from 4 biologic repeats; scale bar: 50 μm. Source data are provided as a Source Data file.

Supplementary Fig. 6

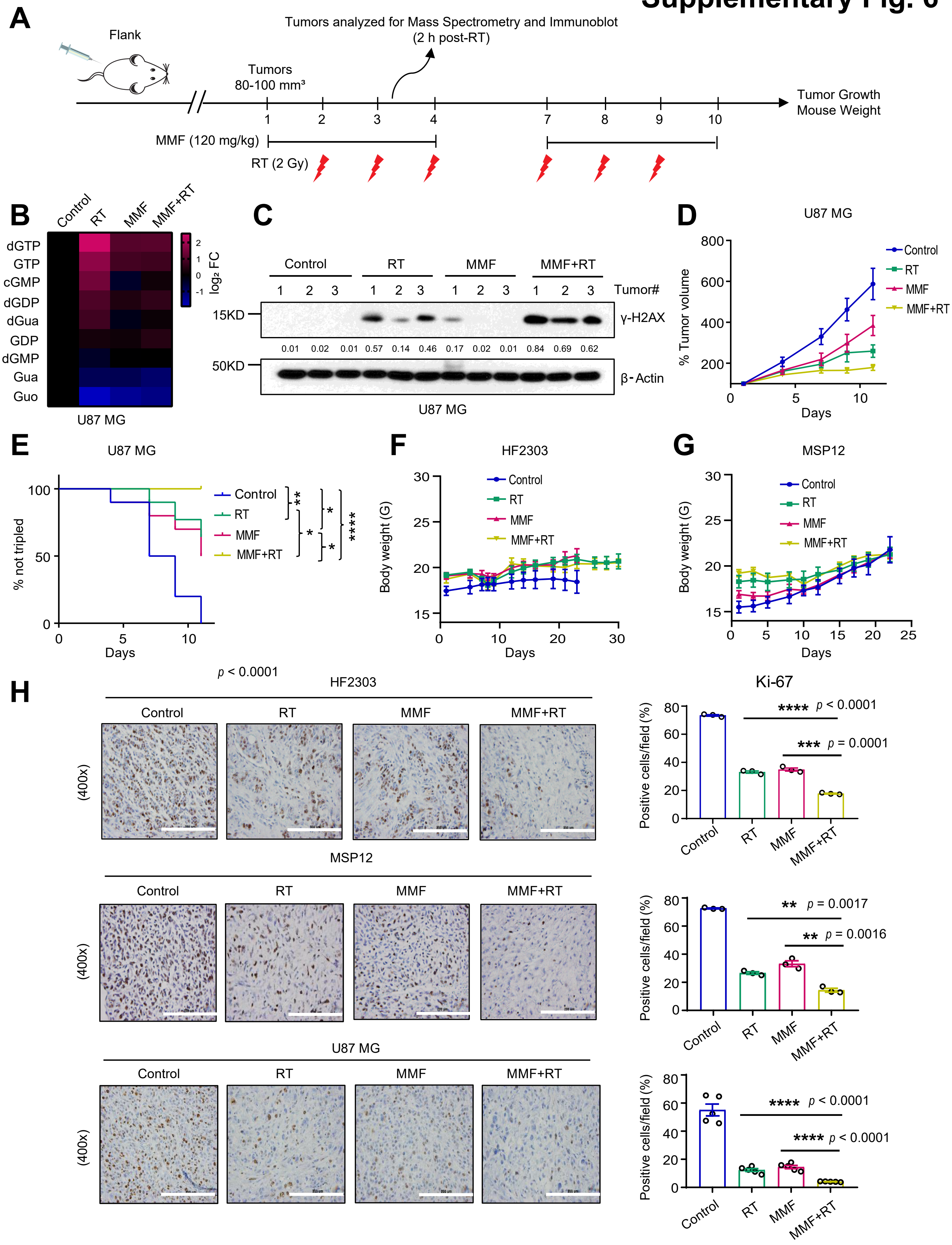

Supplementary Figure 6. MMF augments RT efficacy against immortalized and patient-derived GBM flank xenografts

(A) A schematic timeline of U87 MG flank model. U87 MG cells (2 x 10^6) were injected into flanks and tumors were allowed to form. Mice were randomly divided as described in Methods. MMF (120 mg/kg) was administered via oral gavage once daily 2 h before RT on weekdays (8 doses) and RT (2 Gy/fraction) was administered over 6 daily fractions on weekdays. **(B&C)** Tumors harvested 2 h after receiving the second RT dose were analyzed by LC-MS/MS (B), or were ground and lysed for immunoblotting assay with indicated antibodies (C). The bands of immunoblot were quantified using Image J 2.0 software and the quantified numbers were labeled under each band. Fig. C is one representative figures from 3 biologic repeats**.** **(D)** Tumor volumes for the indicated treatment conditions are normalized to the individual tumor sizes defined on day 1. Error bars indicate mean ± SEM from 10 tumors from 5 mice per group. **(E)** Kaplan-Meier estimates of time to tumor tripling. *p*-value for Control vs. RT is 0.0020; Control vs. MMF is 0.0111; Control vs. MMF+RT is < 0.0001; RT vs. MMF+RT is 0.0444; MMF vs. MMF+RT is 0.0118; *, $p < 0.05$; **, $p < 0.01$; ****, $p < 0.0001$. The *p*-value was obtained by the log-rank (Mantel–Cox) test. **(F&G)** Body weight curve of HF2303 and MSP12 GBM model. Body weight was measured during the treatment and plotted. Data are presented as mean ± SEM from 5 independent mice per group. **(H)** Representative images and analysis of Ki-67 expression in four arms of treatment. Staining quantification: positively stained cells were counted out of 3-6 different fields for each tumor. Data are presented as mean ± SEM from 3 independent tumors per group in HF2303 and MSP12, and mean ± SEM from 5 independent tumors per group in U87. **, $p < 0.01$; ****, $p < 0.0001$; scale bar: 200 μm. The *p*-values indicated in Fig. H were performed by two-tailed unpaired student t test. Source data are provided as a Source Data file.

Supplementary Fig. 7

A

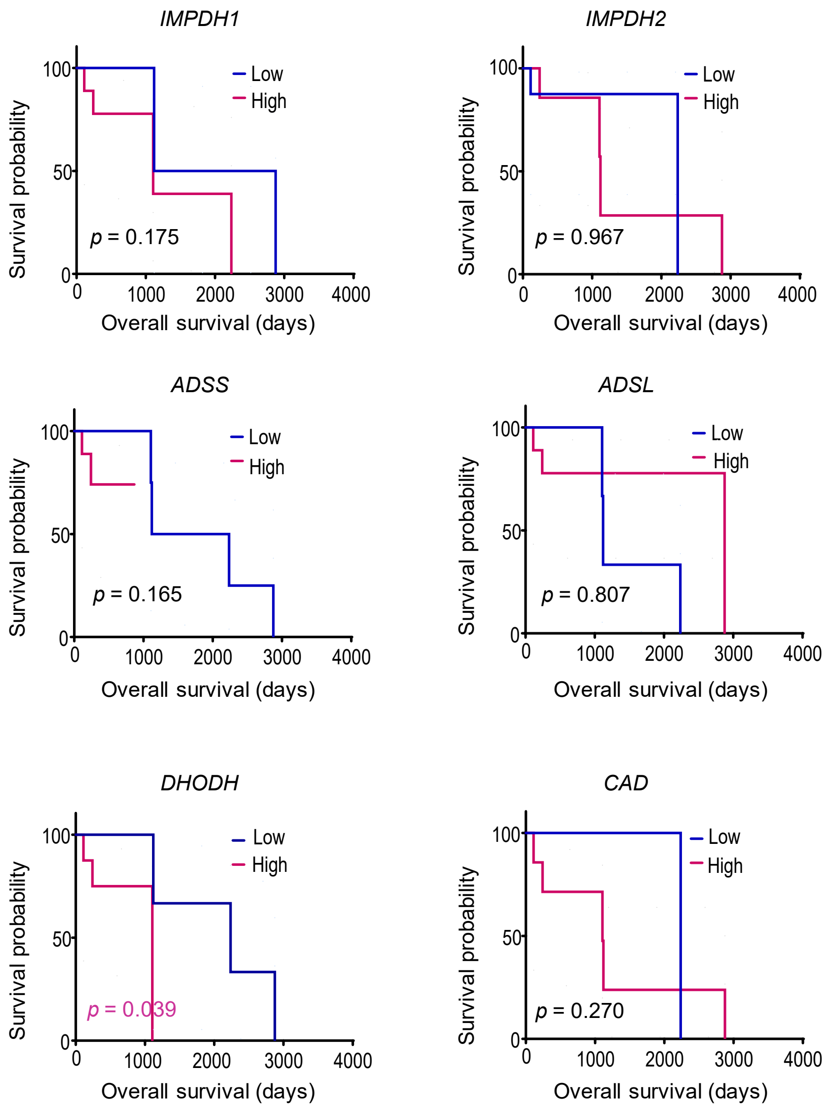

Supplementary Figure 7. Correlation between expression of enzymes involved in nucleotide synthesis and survival in IDH mutant GBMs.

(A) Patients (n = 22) from the TCGA Pan-Cancer Atlas with IDH mutant GBM were stratified by median expression of rate limiting enzymes in nucleotide synthesis and survival was estimated by the Kaplan-Meier method. *p*-values were obtained using the log-rank (Mantel–Cox) test. Source data are provided as a Source Data file.

Supplementary Figure 8. Uncropped Original Scans

Figure 6C

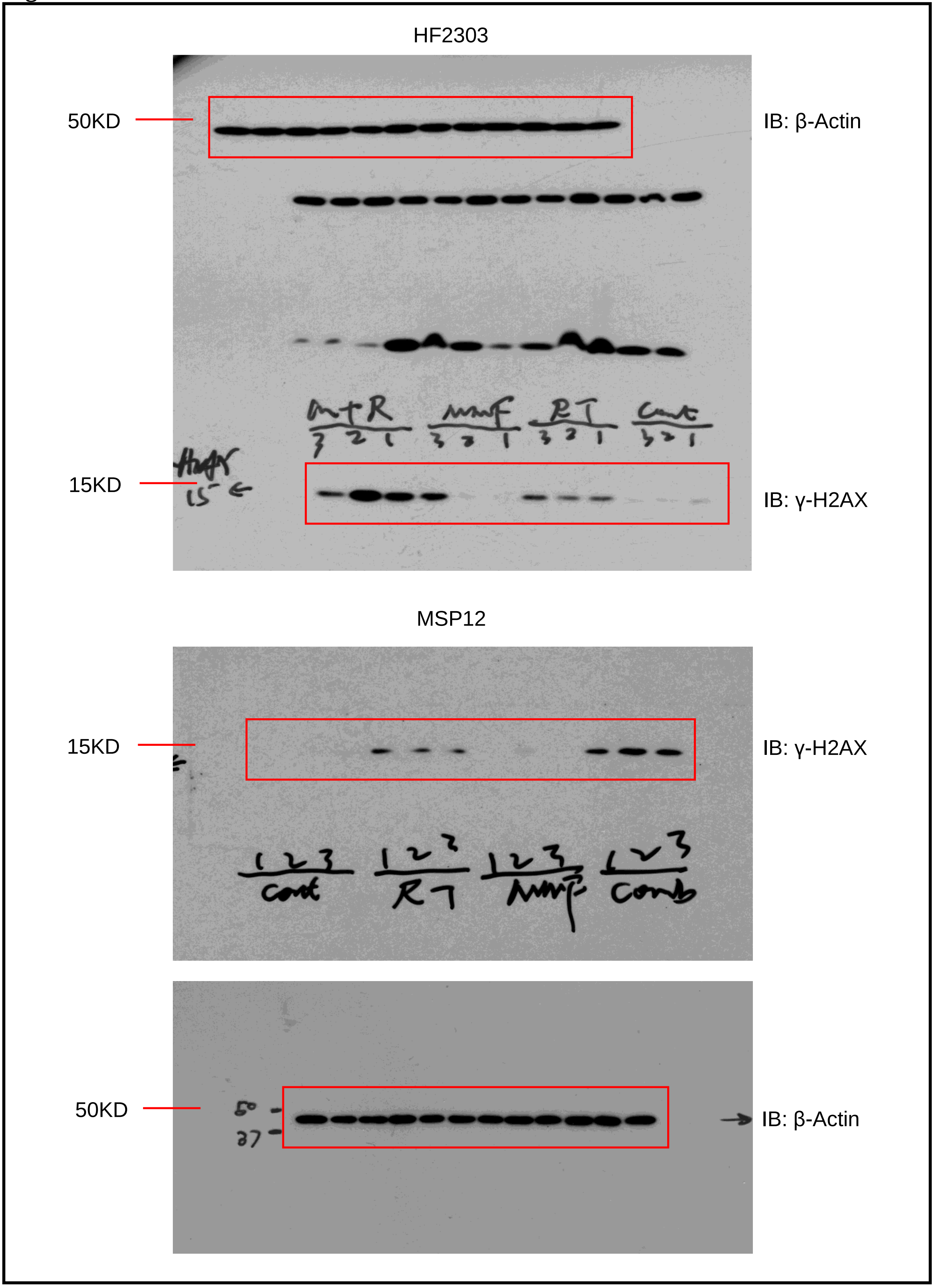

Supplementary Figure 8. Uncropped Original Scans

Supplementary Figure 3G

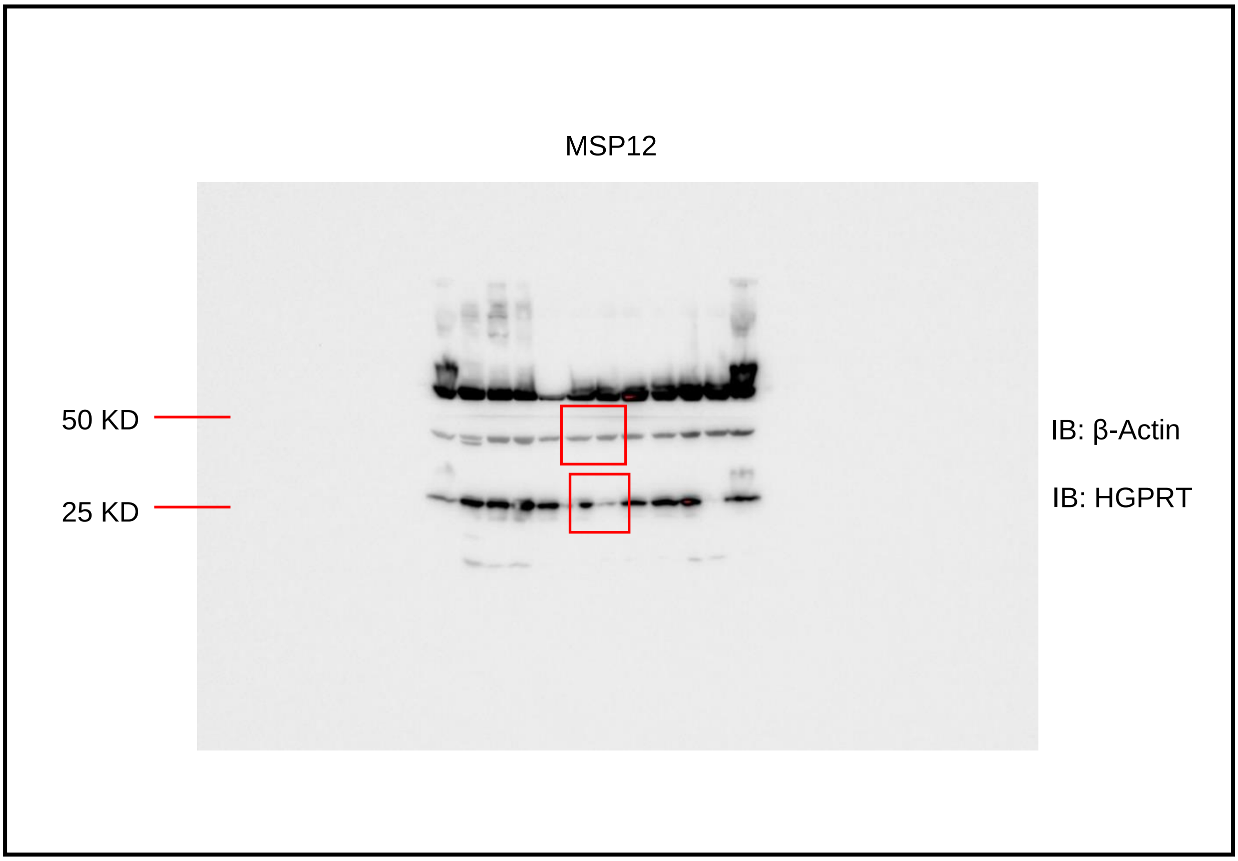

Supplementary Figure 8. Uncropped Original Scans

Supplementary Figure 6C

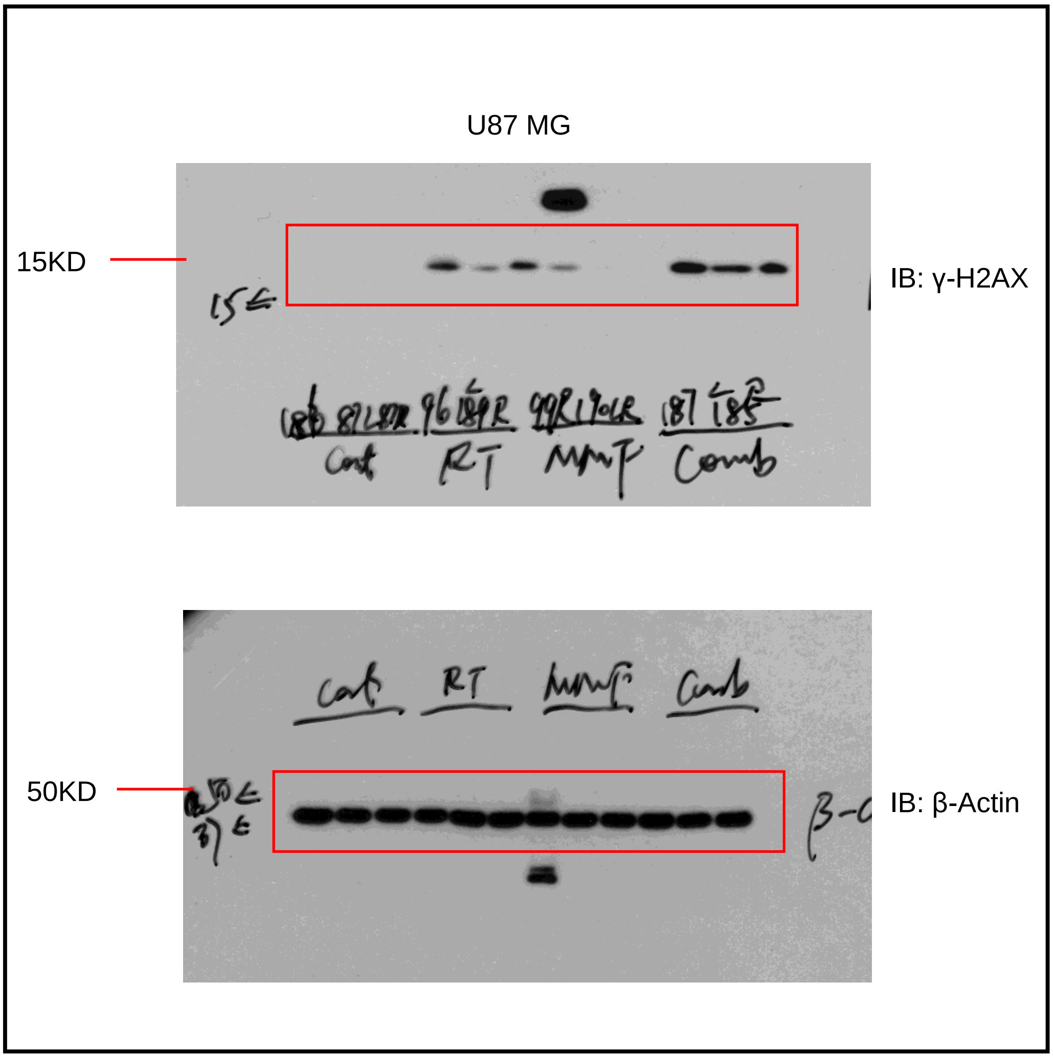

Supplementary Figure 9. Gating information for Figure 1B (analysis of γH2AX after RT)

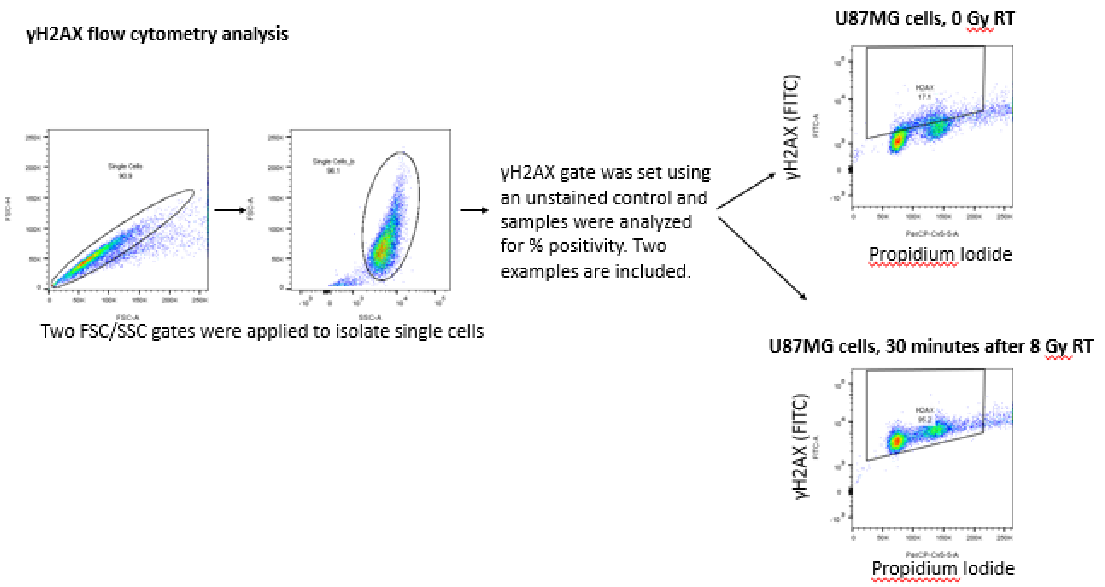

Supplementary Figure 10. Gating information for Supplementary Figure 1B (Cell Cycle Analysis)

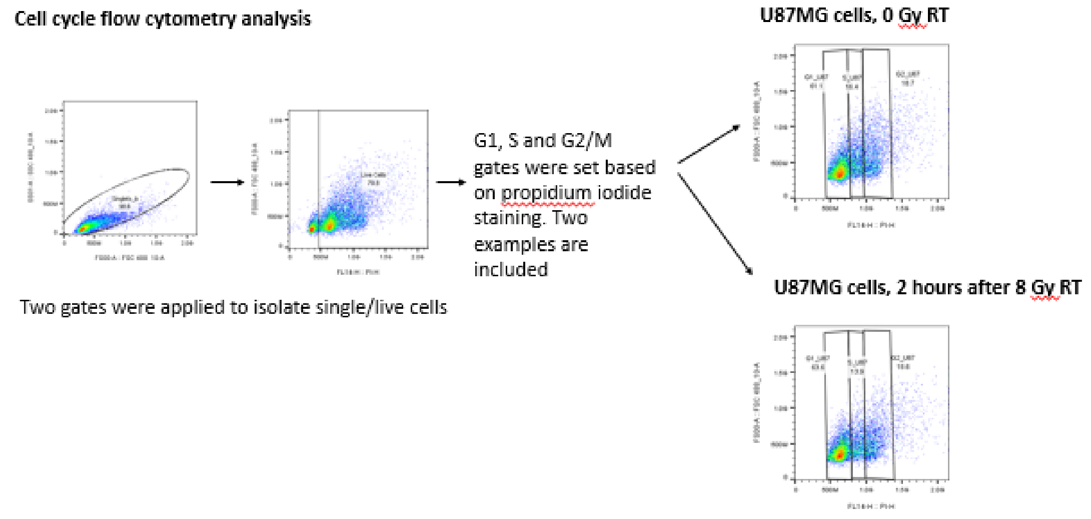

Supplementary Table 1 Details of the GBM cell lines sources

Cell line	Company/Source	Catalog No.
U87 MG	ATCC	HTB-14
A172	ATCC	CRL-1620
DKMG	DSMZ	ACC 277
8MGBA	DSMZ	ACC 432
42MGBA	DSMZ	ACC 431
KNS-60	JCRB	IFO50357
AM-38	JCRB	IFO50492
M059K	ATCC	CRL-2365
KNS-81	JCRB	IFO50359
YKG-1	JCRB	JCRB0746
U138	ATCC	HTB-16
T98G	ATCC	CRL-1690
LN18	ATCC	CRL-2610
SNB19	DSMZ	ACC 325
GOS3	DSMZ	ACC 408
LN229	ATCC	CRL-2611
GMS10	DSMZ	ACC 405
GB-1	JCRB	IFO50489
U118 MG	ATCC	HTB-15
SW1783	ATCC	HTB-13

KS-1	JCRB	IFO50436
DBTRG-05MG	ATCC	CRL-2020

ATCC: American Type Culture Collection

DSMZ: Leibniz Institute DSMZ-German Collection of Microorganisms and Cell Cultures

JCRB: Japanese Collection of Research Bioresources Cell Bank
