# Supplementary material for: Purine metabolism regulates DNA repair and therapy resistance in glioblastoma
Source: Nat Commun. 2020 Jul 30;11:3811. doi: 10.1038/s41467-020-17512-x (PMC7393131; doi:10.1038/s41467-020-17512-x)
Supplement: Supplementary file 3 — Description of Additional Supplementary Information [file 41467_2020_17512_MOESM3_ESM.pdf]

Description of Additional Supplementary Files

File Name: Supplementary Data 1
Description: The metabolomic data of Fig. 1c-e.

File Name: Supplementary Data 2
Description: The metabolomic data of Fig. 3b-d, Fig. 6b and Supplementary Fig. 5b-f, 6b.
