# Supplementary material for: Purine metabolism regulates DNA repair and therapy resistance in glioblastoma
Source: Nat Commun. 2020 Jul 30;11:3811. doi: 10.1038/s41467-020-17512-x (PMC7393131; doi:10.1038/s41467-020-17512-x)
Supplement: Supplementary file 6 — Reporting Summary [file 41467_2020_17512_MOESM6_ESM.pdf]

nature research

Corresponding author(s): Daniel R. Wahl

Last updated by author(s): 06-17-2020

Reporting Summary

Nature Research wishes to improve the reproducibility of the work that we publish. This form provides structure for consistency and transparency in reporting. For further information on Nature Research policies, see Authors & Referees and the Editorial Policy Checklist .

Statistics

For all statistical analyses, confirm that the following items are present in the figure legend, table legend, main text, or Methods section.

n/a	Confirmed	
☐	☒	The exact sample size (*n*) for each experimental group/condition, given as a discrete number and unit of measurement
☐	☒	A statement on whether measurements were taken from distinct samples or whether the same sample was measured repeatedly
☐	☒	The statistical test(s) used AND whether they are one- or two-sided<br>*Only common tests should be described solely by name; describe more complex techniques in the Methods section.*
☐	☒	A description of all covariates tested
☐	☒	A description of any assumptions or corrections, such as tests of normality and adjustment for multiple comparisons
☐	☒	A full description of the statistical parameters including central tendency (e.g. means) or other basic estimates (e.g. regression coefficient) AND variation (e.g. standard deviation) or associated estimates of uncertainty (e.g. confidence intervals)
☐	☒	For null hypothesis testing, the test statistic (e.g. *F*, *t*, *r*) with confidence intervals, effect sizes, degrees of freedom and *P* value noted<br>*Give P values as exact values whenever suitable.*
☒	☐	For Bayesian analysis, information on the choice of priors and Markov chain Monte Carlo settings
☒	☐	For hierarchical and complex designs, identification of the appropriate level for tests and full reporting of outcomes
☐	☒	Estimates of effect sizes (e.g. Cohen's *d*, Pearson's *r*), indicating how they were calculated

Our web collection on statistics for biologists contains articles on many of the points above.

Software and code

Policy information about availability of computer code

Data collection	1. Clonogenic survival, γ-H2AX foci formation, comet assay, Ki-67 IHC staining, and metabolite level analysis after MPA and teriflunomide treatment were analyzed by unpaired two-tailed t tests using GraphPad Prism Version 8.<br>2. The correlation between metabolites and RT-resistance score were analyzed using Pearson's linear correlation function in MATLAB.<br>3. All chromatography analysis was done with Agilent MassHunter Quantitative Analysis 9.0.647.0.<br>4. The flow data in this manuscript were analyzed using FlowJo 7.6 software (Three Star).
Data analysis	1. The correlated pathways shown in Fig. S1H were visualized on a human metabolic network map using the iPath3.0: interactive pathways explorer v3 (Nucleic Acids Res. 2018 Jul 2;46(W1):W510-W513).<br>2. The Cancer Genome Atlas (TCGA) PanCancer Atlas LGG and GBM cohorts were used for survival analysis and gene expression profiling in Fig. 7G (Cell 173, 400-416 e411 (2018)). We excluded cases based on those that were masked ("Do_not_use") according to the Pan-Cancer Atlas sample quality annotations (http://api.gdc.cancer.gov/data/1a7d7be8-675d-4e60-a105-19d4121bdebf).

For manuscripts utilizing custom algorithms or software that are central to the research but not yet described in published literature, software must be made available to editors/reviewers. We strongly encourage code deposition in a community repository (e.g. GitHub). See the Nature Research guidelines for submitting code & software for further information.

Data

Policy information about availability of data

All manuscripts must include a data availability statement. This statement should provide the following information, where applicable:

- Accession codes, unique identifiers, or web links for publicly available datasets
- A list of figures that have associated raw data
- A description of any restrictions on data availability

1. Data availability

Source data underlying Figs. 1a-e, 2b-i, 3b-h, 4a-f, 5a-g, 6b-g, 7c-e and Supplementary Figs. 1a, b, f, g, 3a-d, f-h, 5b-f, 6b-h, and 7a are provided as a Source Data file. The metabolomic data of Fig. 1c-e (Supplementary Data 1), and Fig. 3b-d, Fig. 6b and Supplementary Fig. 5b-f, 6b (Supplementary Data 2) for this article are available as Supplementary Information files. Further data are available from the corresponding author upon reasonable request.
2. The Cancer Genome Atlas (TCGA) PanCancer Atlas LGG and GBM cohorts were used for survival analysis and gene expression profiling in Fig. 7G (Cell 173, 400-416 e411 (2018)). We excluded cases based on those that were masked ("Do_not_use") according to the Pan-Cancer Atlas sample quality annotations (http://api.gdc.cancer.gov/data/1a7d7be8-675d-4e60-a105-19d4121bdebf).

Field-specific reporting

Please select the one below that is the best fit for your research. If you are not sure, read the appropriate sections before making your selection.

[x] Life sciences [ ] Behavioural & social sciences [ ] Ecological, evolutionary & environmental sciences

For a reference copy of the document with all sections, see nature.com/documents/nr-reporting-summary-flat.pdf

Life sciences study design

All studies must disclose on these points even when the disclosure is negative.

Sample size

1. For the in vitro assays (cell line level), we lacked a priori effect size estimates. We thus could not perform a sample size calculation. Rather, we performed at least n=2-3 biological repeats with 2-3 technical repeats for each experiment, which is standard way to proceed with in vitro experiments in the absence of known effect size.
2. For the in vivo xenograft and intracranial survival analysis (mouse level), we did a sample size calculation. A two-sided log rank test with an overall sample size of 5 subjects in each group could achieve 90% power at a 0.05 significance level.

Data exclusions

No relevant data has been excluded from the analysis.

Replication

We made duplicate, or triplicate, or quadruplicate, or sextuplicate (like celltiter-glo assay) for each condition and repeated 3-5 times for each experiment and the reproducibility of all the data shown in the current study is successful.

Randomization

All the samples (like mouse or mouse tumors) were randomly allocated into experimental groups.

Blinding

The investigators were all blinded to group allocation during data collection and analysis. For some experiments where the investigators were physically placing mice in the irradiator, they could not be blinded to the groups.

Reporting for specific materials, systems and methods

We require information from authors about some types of materials, experimental systems and methods used in many studies. Here, indicate whether each material, system or method listed is relevant to your study. If you are not sure if a list item applies to your research, read the appropriate section before selecting a response.

Materials & experimental systems

n/a	Involved in the study	
[ ]	[x]	Antibodies
[ ]	[x]	Eukaryotic cell lines
[x]	[ ]	Palaeontology
[ ]	[x]	Animals and other organisms
[x]	[ ]	Human research participants
[x]	[ ]	Clinical data

Methods

n/a	Involved in the study	
[x]	[ ]	ChIP-seq
[ ]	[x]	Flow cytometry
[x]	[ ]	MRI-based neuroimaging

Antibodies

Antibodies used

1. Anti-phospho-Histone H2AX (Millipore, Cat # 05-636, Clone JBW301, Lot # 3108494)
2. β-actin (Cat# 4967, Cell Signaling Technology, Lot # D0218).
3. Ki-67 antibody (BD Biosciences, Cat # 550609, Lot # 8158790)
4. HGPRT antibody (PA5-22281, Thermo Fisher Scientific)
5. FITC conjugated anti-mouse secondary antibody (Cat# A11005, Invitrogen, Lot # 1937185)

Validation

1. Anti-phospho-Histone H2A.X (Ser139)
Source: Clone JBW301 is a well published Mouse Monoclonal Antibody
Species Reactivity: Vertebrates
Application: validated in ChIP, ICC, IF, WB. This purified mAb is highly specific for phospho-Histone H2A.X (Ser139) also known as H2AXS139p.

2. β-actin antibody
Source: Rabbit.
Species Reactivity: Human, Mouse, Rat, Hamster, Monkey, Mink, D. melanogaster, Zebrafish, Bovine
Application Key: W-Western, IP-Immunoprecipitation, IHC-Immunohistochemistry, ChIP-Chromatin Immunoprecipitation, IF-Immunofluorescence, F-Flow Cytometry, E-P-ELISA-Peptide.
3. Ki-67 antibody
Source: Mouse IgG1, κ
Species Reactivity: Human (QC Testing) Mouse (Tested in Development) Rat, Rhesus (Reported)
Application: Flow cytometry (Routinely Tested), Immunohistochemistry-frozen, Immunohistochemistry-formalin (antigen retrieval required) (Tested During Development)
4. HGPRT antibody
Species reactivity: Human, Mouse, Rat
Host/Isotype: Rabbit/IgG
Class: polyclonal
Application:Immunocytochemistry, immunofluorescence, immuohistochemistry, westen blot
5. FITC conjugated anti-mouse secondary antibody
Species Reactivity: Mouse
Host /Isotype: Goat/IgG
Class: Polyclonal
Type: Secondary Antibody
Application: Flow cytometry, Immunocytochemistry, immunofluorescence, immuohistochemistry

Eukaryotic cell lines

Policy information about cell lines

Cell line source(s)

1. U87 MG, A172, M059K, U138, T98G, LN18, LN229, U118 MG, SW1783, and DBTRG were obtained from American Type Culture Collection (ATCC).
2. DKMG, 8MGBA, 42MGBA, SNB19, GOS3, and GMS10 were purchased from Leibniz Institute DSMZ-German Collection of Microorganisms and Cell Cultures.
3. KNS-60, AM-38, KNS-81, YKG-1, GB-1, and KS-1 were purchased from Japanese Collection of Research Bioresources Cell Bank (JCRB).
4. HF2303 primary neurosphere, which was originally described by Dr. Tom Mikkelsen at Henry Ford Hospital (Detroit, MI) (Oncotarget. 2016;7(39):63020-41), was a kind gift from Dr. Alnawaz Rehemtulla and MSP12 was a gift from Drs. Pedro Lowenstein and Maria Castro. GBM38, which is an intrinsically RT-resistant PDX model of primary GBM from the Mayo Clinic Brain Tumor Patient-Derived Xenograft National Resource, was kindly provided by Dr. Jann Sarkaria (Zhao et al., Neuro Oncol. 2019 May 23).

Authentication

Cell line authentication was performed by the originating cell line repositories and then used immediately upon receipt. Cell lines were re-authenticated using short tandem repeat profiling if they had been using for longer than 1 year.

Mycoplasma contamination

Mycoplasma test (Cat# LT07-418, Lonza) were performed monthly in our lab and all the cell lines used during the study were mycoplasma negative.

Commonly misidentified lines
(See ICLAC register)

No commonly misidentified cell line was used in the study.

Animals and other organisms

Policy information about studies involving animals; ARRIVE guidelines recommended for reporting animal research

Laboratory animals

C.B-17 SCID mice (female, 4-7 weeks old) were obtained from Envigo and maintained in specific pathogen-free conditions. 6-week-old male and female Rag1-KO mice (B6.129s7-RAG1 tm/Mom/J) were obtained from the Jackson Laboratory. The mice were housed in accordance to the University of Michigan Institutional Animal Care & Use Committee (IACUC) rules and guidelines. University of Michigan's Unit for Laboratory Animal Medicine (ULAM) ensured that the housing temperature was kept at 74 degrees F, relative humidity between 30%-70%, and was on a light/dark cycle of 12 hours on/12 hours off.

Wild animals

No wild animals were used in the study.

Field-collected samples

No field collected samples were used in the study.

Ethics oversight

All mouse experiments were approved by the University Committee on Use and Care of Animals at the University of Michigan.

Note that full information on the approval of the study protocol must also be provided in the manuscript.

Flow Cytometry

Plots

Confirm that:

- [x] The axis labels state the marker and fluorochrome used (e.g. CD4-FITC).
- [x] The axis scales are clearly visible. Include numbers along axes only for bottom left plot of group (a 'group' is an analysis of identical markers).
- [x] All plots are contour plots with outliers or pseudocolor plots.
- [x] A numerical value for number of cells or percentage (with statistics) is provided.

Methodology

Sample preparation	1. For cell cycle analysis in Figure S1B, cells were harvested and fixed with pre-cooled 70% ethanol overnight, followed by staining with propidium iodide/RNase A staining buffer in the dark at room temperature for 15 min.<br>2. For the r-H2AX analysis shown in Figure 1B, cells were fixed in ice-cold 70% ethanol, and incubated with a mouse monoclonal anti-phospho-Histone γ-H2AX antibody and with a FITC conjugated anti-mouse secondary antibody, followed by staining with propidium iodide/RNase A to assess total DNA content.
Instrument	Samples were analyzed on a FACScan flow cytometer (Becton Dickinson).
Software	The data were analyzed with FlowJo software 7.6 (Tree Star).
Cell population abundance	Cells were not sorted in this study.
Gating strategy	For quantification of r-H2AX positivity, a gate was arbitrarily set on the control, untreated sample to define a region of positive staining for gH2AX of approximately 5% to 10%. This gate was then overlaid on the treated samples.

- [x] Tick this box to confirm that a figure exemplifying the gating strategy is provided in the Supplementary Information.
